# Tuberculosis of the Left Wrist Joint and Spine

**DOI:** 10.7759/cureus.6203

**Published:** 2019-11-19

**Authors:** Parthiban Sivasamy, Mohd Yazid Bajuri, Abdul Wahab Ghani

**Affiliations:** 1 Orthopaedics, KPJ Healthcare University College, Nilai, MYS; 2 Orthopaedics and Traumatology, Universiti Kebangsaan Malaysia, Kuala Lumpur, MYS; 3 Orthopaedics, Ampang Hospital, Kuala Lumpur, MYS

**Keywords:** tuberculosis, wrist arthritis, wrist tuberculosis, tubercular arthritis, septic arthritis

## Abstract

The incidence of wrist tuberculosis is rare. Clinical features and radiographs are not conclusive in the beginning, happen to delay the achievement of the diagnosis, and thus result in poor treatment. We present a case report of wrist tuberculosis that had delayed diagnosis. Hence, the initiation of antituberculous treatment was delayed, as the initial investigations were not conclusive of tuberculous infection. The patient was treated surgically multiple times before tuberculosis was diagnosed. Antituberculous chemotherapy was started for the patient for one year until she became afebrile and infective markers returned to normal. However, the patient developed left wrist stiffness due to arthrofibrosis and bony destruction of the wrist joint.

## Introduction

Skeletal involvement occurs in 1% to 3% of overall patients with tuberculosis (TB) [1]. It is estimated that 50% of skeletal TB is of the spine and extraspinal articulating joints [2]. The wrist joint is among the rare sites. TB of the joints has a prolonged onset and is rarely diagnosed before developing into severe arthritis [3].

Poncet’s disease or TB rheumatism is differentiated from TB arthritis, as the former disease is a non-destructive variant of joint inflammation, which is observed during the acute phase of TB. TB arthritis commonly affects a single joint and is where the organism can be obtained [4]. TB arthritis begins with synovitis, leads to periarticular demineralization, subsequently to marginal erosions, and eventually to damage the joint [5]. Quicker progression from synovial inflammation damage of the joint is seen in weight-bearing patients. In the presence of a superinfection, i.e. Staphylococcus aureus, the acceleration of joint destruction occurs along with a systemic inflammatory response [2].

The delay in diagnosis is contributed by a non-directional misleading presentation of patients such as malaise and constitutional symptoms [2]. These delays in mycobacterial infection diagnosis establishment and delayed initiation of treatment could result in the destruction of more portions of the bone, adjacent bones, or joints [5]. Therefore, tubercular arthritis is to be understood clearly by its manifestation, diagnosis establishment, and treatment.

## Case presentation

A 70-year-old woman, admitted on May 26, 2017, to the orthopedic department, presented with symptoms of left wrist pain and swelling, associated with fever in the past two days. The swelling was significant enough to expand over the palmar and dorsal aspect of the hand. It was soft on palpation, with minimal fluctuation, mild edema over the dorsum, tender, and warm. She had underlying diabetes mellitus and ischemic heart disease. There was no past or family history of TB. She had no complaints of respiratory symptoms or hemoptysis.

**Figure 1:**
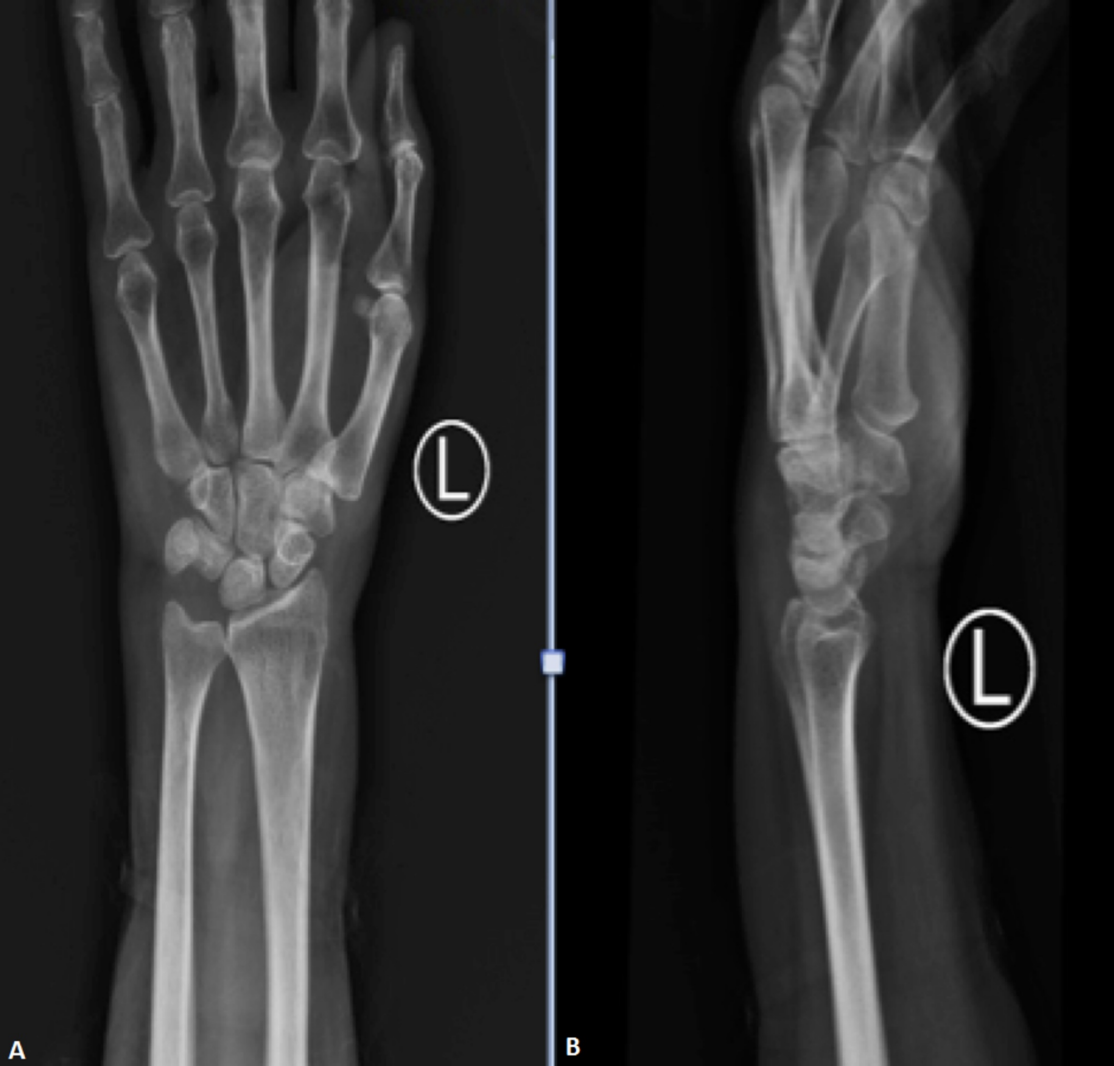
X-ray film of the left wrist on admission; increased soft tissue shadow with no obvious abnormality A: antero-posterior view; B: lateral view

Blood investigations revealed a total white cell count (TWC) of 38,000/UL, erythrocyte sedimentation rate (ESR) of 45 mm/h, and C-reactive protein (CRP) of 455 mg/L. The blood culture and sensitivity revealed Streptococcus pyogenes. A left wrist X-ray was performed with no abnormal findings (Figures 1A-1B). The chest radiograph was normal.

**Figure 2:**
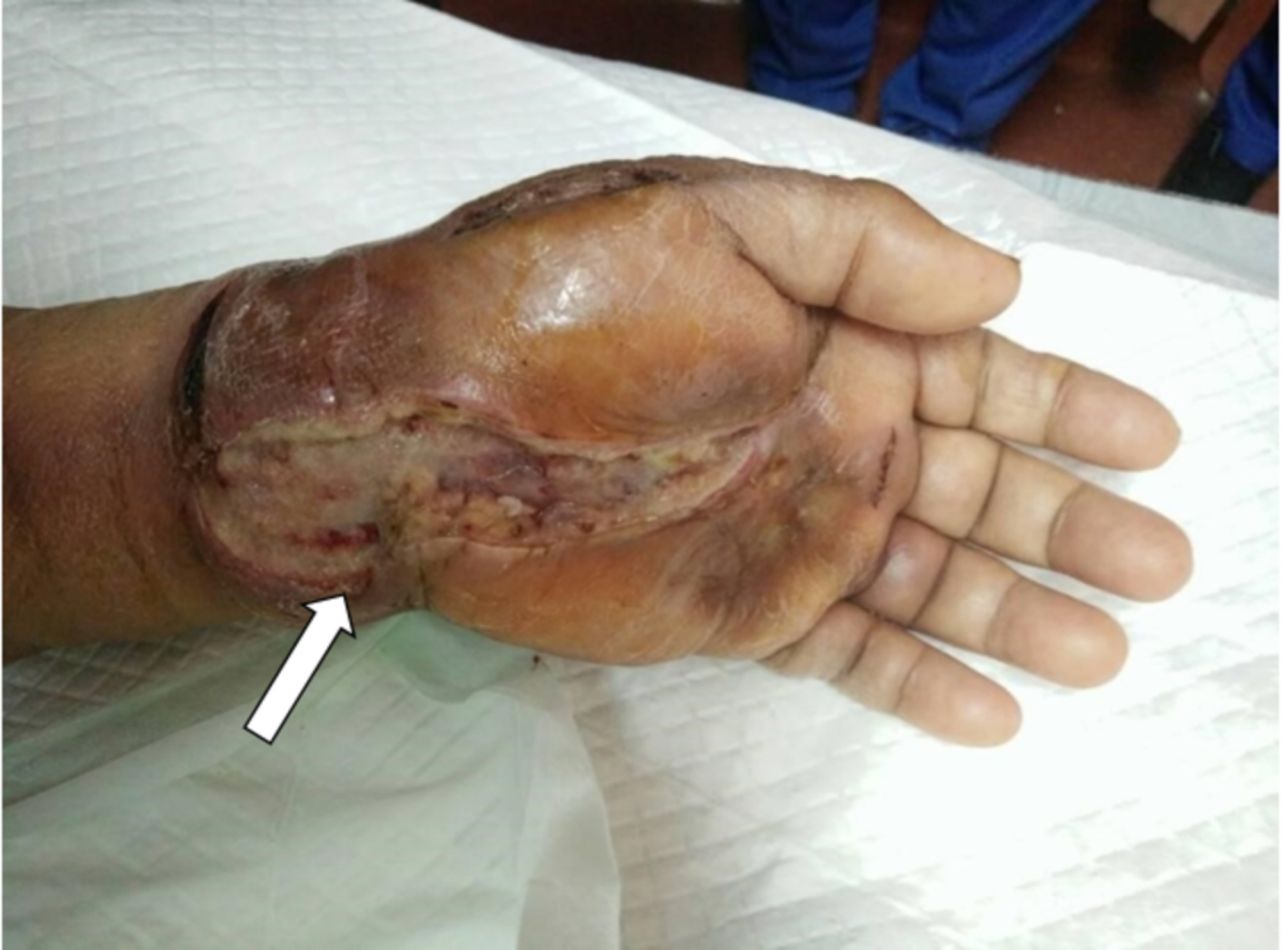
Swollen left hand picture taken after the first drainage and debridement

She was diagnosed with a left wrist abscess and a left wrist incision and drainage with flexor retinaculum release was performed (Figure 2).

A dense yellow fluid was seen during the operation. Intraoperative specimens were sent to microbiology. The pus culture and sensitivity were negative. Based on blood culture and sensitivity, a Streptococcus pyogenes antibiotic therapy, intravenous (IV) meropenem 1 g tds was administered over a course of four weeks, and discharged with another two weeks of oral unasyn 375 mg bd. The white blood cell (WBC), C-reactive protein (CRP), and erythrocyte sedimentation rate (ESR) were all normal readings by the end of six weeks of IV meropenem.

**Figure 3:**
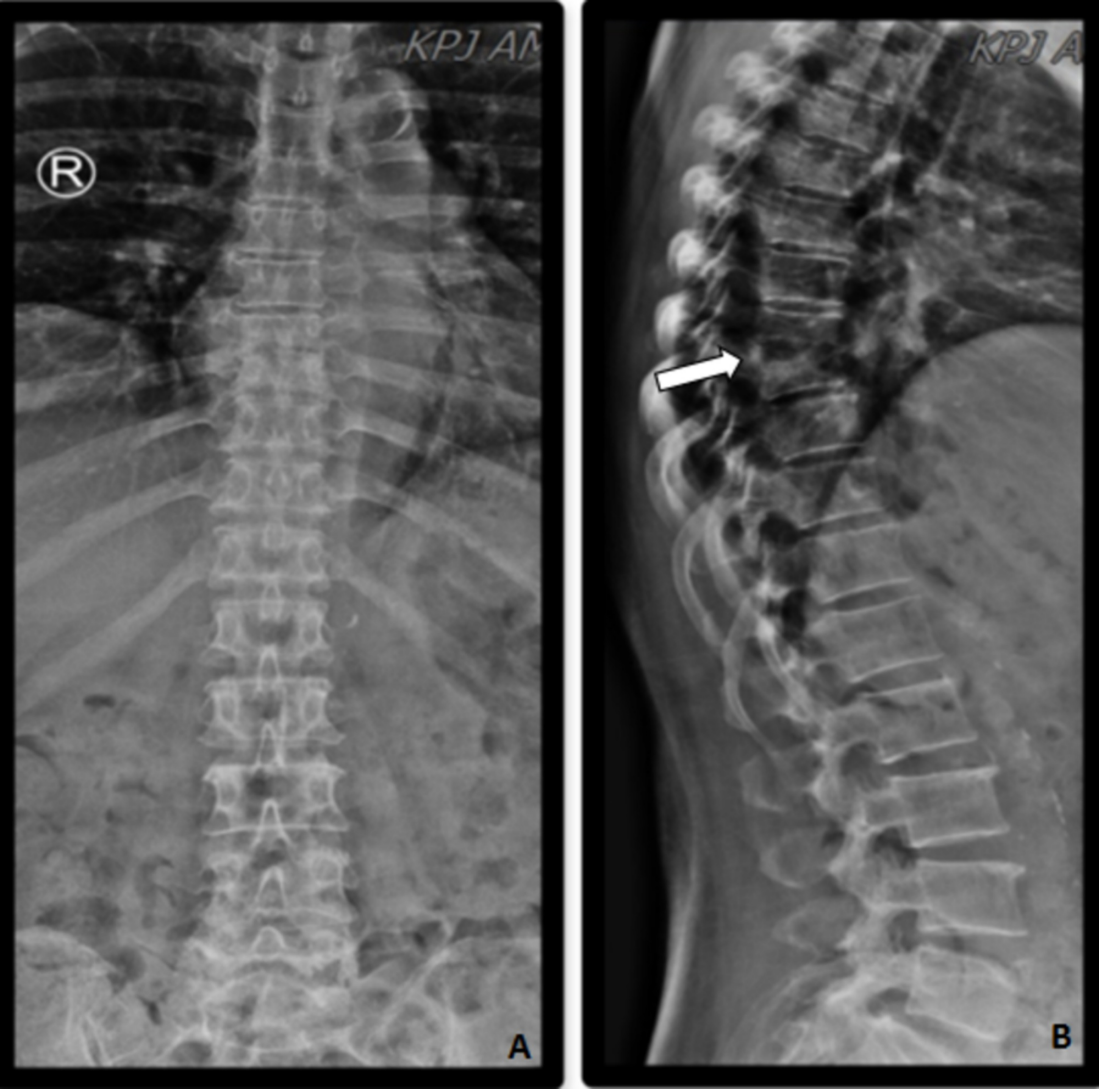
X-ray film of the spine at the two months follow-up. There is reduction of T8 and T9 vertebral body height, erosion of the superior endplate of T9 noted as shown in Figure 2B. The T8/T9 intervertebral disc space is narrowed. Appearance suggestive of discitis with paravertebral soft tissue collection at T8/T9 level and erosion of the T8 and T9 vertebra. A : antero-posterior view; B : lateral view

However, the wrist was warm and she developed recurrent swelling in the course of six weeks after discharge. She also complained of back pain. A thoracolumbar X-ray revealed erosion of the T8 and T9 vertebral bodies (Figures 3A-3B).

**Figure 4:**
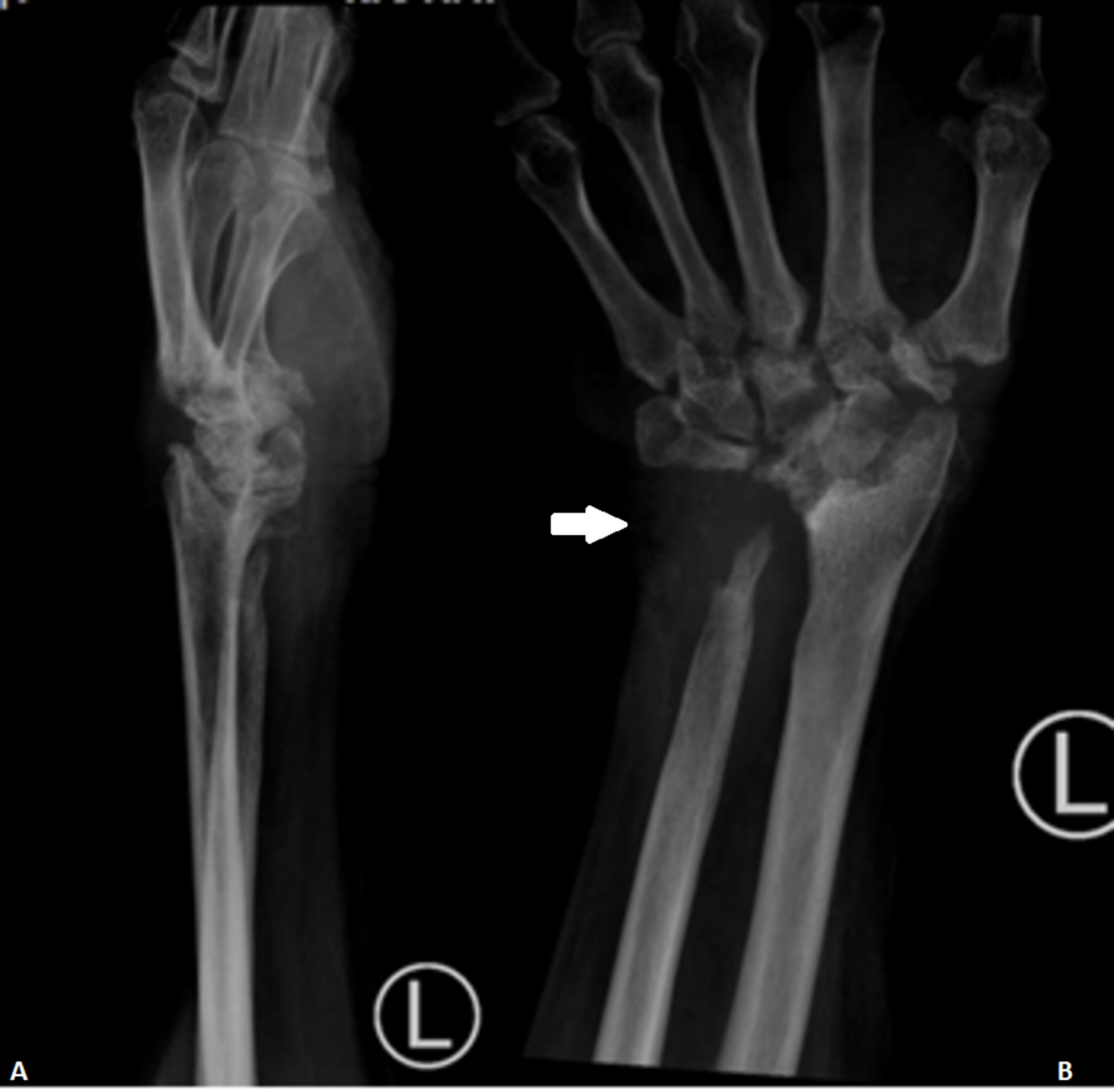
X-ray film of the left wrist after one year of antituberculous therapy. Based on Figure 4B, there is erosion and a lytic lesion around the bony skeleton at the wrist. Mild reduction of the bony density is seen at the carpals and metacarpals. A : lateral view; B : antero-posterior view

The repeated left wrist X-ray revealed erosive arthritis of the wrist and carpal joints (Figures 4A-4B).

**Figure 5:**
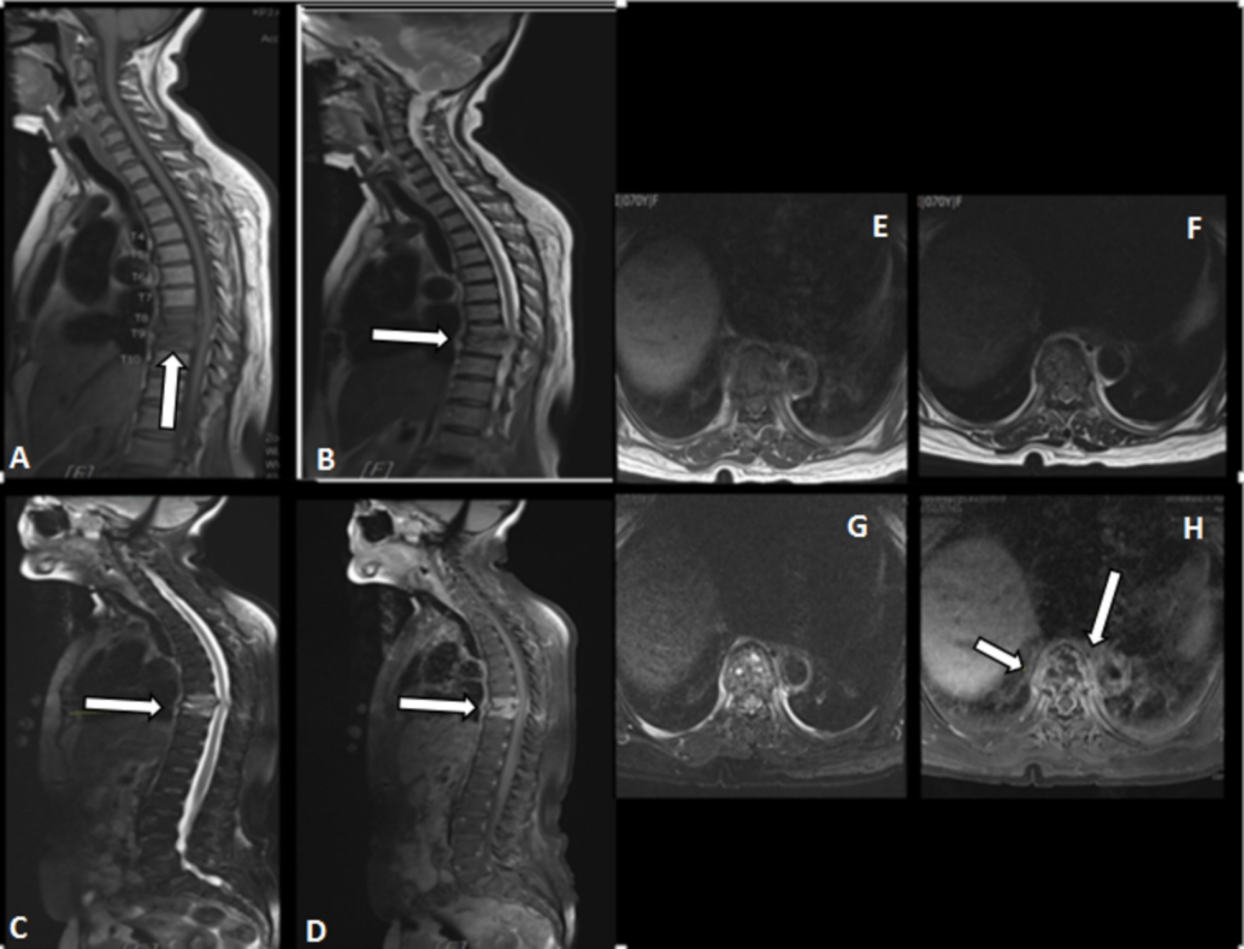
MRI films of the spine at the two months follow-up, suggestive of spondylodiscitis of T8 and T9 with suspicion of tuberculosis infection A-D : sagittal view; E-H : axial view

Magnetic resonance imaging (MRI) thoracolumbar done was suggestive of spondylodiscitis of T8 and T9, with suspicion of tuberculosis infection (Figures 5A-5H).

**Figure 6:**
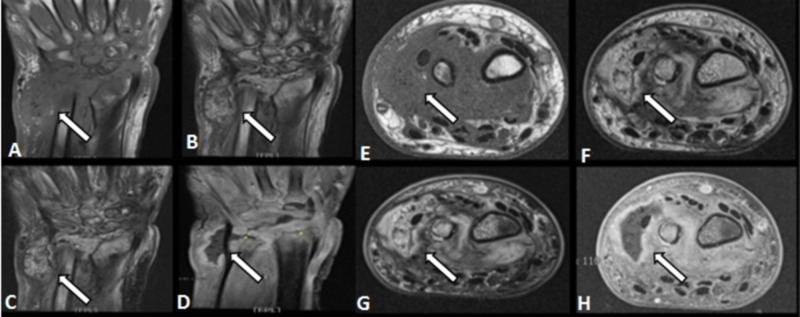
MRI film of the left wrist at the two months follow-up, suggestive of erosive arthritis with a gas-forming abscess along the ulnar side A-D : coronal view; E-H : axial view MRI : magnetic resonance imaging

MRI of the left wrist was suggestive of erosive arthritis with a gas-forming abscess along the ulnar side. It showed fluid collection in the intraarticular and extraarticular spaces of the wrist (Figures 6A-6H).

The involvement of the spine in diagnostic imaging led to the suspicion of tuberculous infection. Left wrist debridement and arthrotomy were performed and samples sent for biopsy. A spine T8 and T9 transpedicular biopsy was performed.

The blood markers were TWC 10,000/UL, ESR 70 mm/h, and CRP 46 mg/L. The Mantoux test, serum polymerase chain reaction (PCR) tuberculosis (TB), and Quantiferon TB Gold test were performed and the results are negative. The left wrist tissue acid-fast Bacilli (AFB) was revealed as positive during the second-look debridement on October 5, 2017.

She underwent antituberculous therapy for a duration of one year after being diagnosed with left wrist and spine TB. Her left wrist swelling and back pain started to reduce as the antituberculous treatment was initiated and then resolved completely by the end of treatment. However, she developed left wrist stiffness due to arthrofibrosis and bony destruction of the left wrist joint.

## Discussion

TB has always remained a significant infection in developing countries. There are a number of clinical and radiographic features of osseous TB, which mimics a wide range of pathologies, hence, the involvement of extrapulmonary features can be particularly difficult to diagnose [6]. Apart from this, there are also numerous possible differential diagnoses linked, such as subacute or chronic suppurative arthritis, rheumatoid arthritis, benign bone tumors, osteochondrosis, and Kaposi sarcoma, which further makes it difficult to come to a conclusive diagnosis [7]. The lesion also can be misdiagnosed as chronic osteomyelitis as presented by this case in which bone destruction is present. The treatment for cases that involve bone destruction is the eradication of infection by debridement and the usage of antibiotic cement spacer if there is a defect [8]. In our case, it presented as a wrist abscess. Sometimes, if there is a concomitant infection that came before the diagnosis was made, the use of an antibiotic-impregnated medium is very effective, as it delivers high concentrations of antibiotics suffice to treat the local infection site [9].

Articular TB is a chronic and worsening disease that often affects the load-bearing joints. Small joint presentations are not common and diagnosis is usually delayed due to low suspicion. For extrapulmonary involvement, osteoarticular TB is in the fourth position after vertebral, urogenital, and ganglionic localization. Vertebral localization remains the most frequent one [10]. Primary foci reactivation and secondary spreading via the bloodstream are the two gateways to extrapulmonary TB [11]. The discovery of spine involvement two months later in our patient questions which could be the primary foci.

The most common type of presentation of hand TB is tenosynovitis [11]. Tubercular tenosynovitis usually is of gradual onset in nature and progresses slowly. Generally, it is presented with swelling accompanied by mild pain and limitation of movement in the affected area. The swelling will progress through tendons mostly without any other systemic evidence of TB. The most common findings are compound palmar or dumbbell ganglion of the ulnar bursa, “sausage digit,” and carpal tunnel syndrome [11].

Wrist joint TB often presents as swelling without pain that tracks along tendons and is rarely associated with systemic symptoms [1]. However, our case presented with painful progressive swelling, with no involvement of the tendons along with constitutional symptoms.

To confirm the diagnosis of TB, as per the gold standard, the histological pattern of the tissue specimen or any body fluid is needed to detect the presence of acid-fast Bacillus (AFB) [2]. A classic triad of radiological findings, known as the Phemister’s triad, which includes the presence of juxta-articular osteoporosis, peripheral bony erosions, and gradual joint space narrowing suggests the presence of tubercular arthritis. However, such a typical triad may not be seen all the time. Hence, there are high chances of early stages of tubercular arthritis being overlooked by clinicians due to its non-specific clinical signs [12]. In cases where a bone scan was to be performed, it may show in the increased uptake, but this is not specific.

The other radiological examination is MRI, which may showcase the synovial inflammation, fluid collection, bony erosions, and bony lesion. Thus, MRI is useful in revealing the severity of the disease and could be repeated to follow the progress. However, the MRI is nonspecific. The Mantoux test or tuberculin skin test (TST) is used as a supportive examination. Interferon-gamma release assays (IGRA), such as Quantiferon-TB Gold, is specific to detect TB infections but unable to differentiate between actively ongoing disease and latent infection of TB [2].

PCR tests are highly sensitive by detecting amplified TB DNA but unable to distinguish live bacilli from dead bacilli. PCR turns up to 50% to 60% positive in culture-negative groups of cases [2]. The PCR test is specific and faster for obtaining an analysis of synovial fluid, bone, and soft tissue of joints [4].

A synovial biopsy is the current gold standard as a diagnostic test for tubercular arthritis. Approximately, 80% of cases are detected via synovial biopsy, which gives accurate characteristics of lymphocytes, caseating granulomas, and giant cells with caseation.

The treatment is easy, from the three classic shutters of the treatment of osteoarticular TB; only chemotherapy is essential [11]. Chemotherapy has been replaced in recent years with progressively shorter treatment (six months) using more effective drugs [11]. The orthopedic wrist splint maintained until the disappearance of clinical signs (three to four weeks) and followed by rehabilitation [11]. The medical treatment of this type of localized TB mainly consists of anti-bacillary chemotherapy for 12 months, associating isoniazid, rifampicin, pyrazinamide, and ethambutol. Other than the biopsy, there is very little need for surgery.

To date, the treatment options remain debatable. According to Tuli et al., surgical debridement remains a controversial option for wrist joint TB and, in fact, the optimal duration of treatment with antitubercular chemotherapy has been an issue of considerable debate. However, if chemotherapy and debridement are opted for, chemotherapy must be provided prior to any surgical debridement in order to prevent bony destruction and dissemination of disease [13]. A prolonged course of nine or 12 months of multiple chemotherapies should be continued [13].

Atypical site, clinical presentation, and delayed biopsy in our case had delayed the diagnosis of wrist TB. If the wrist TB diagnosis was made early, antituberculous chemotherapy would have been initiated earlier and would have thus reduced the need for repeated surgical debridement and improved the clinical outcome.

## Conclusions

In conclusion, this case reiterates the point that TB can be a great mimic, which can cause a possible deep infection if appropriate treatment is not warranted on time. Keeping this in mind, a clinician should always approach any acute or chronic lesions at the wrist joint with a high index of suspicion, as TB arthritis is uncommon and easily missed. However, in endemic areas, even if other organisms are grown (which was the main red herring in this case), spinal TB led to the correct diagnoses.
